# Combined Physical–Cognitive Therapies for the Health of Older Adults with Mild Cognitive Impairment: A Systematic Review and Meta-Analysis

**DOI:** 10.3390/healthcare13060591

**Published:** 2025-03-08

**Authors:** Juan Miguel Muñoz-Perete, María del Carmen Carcelén-Fraile, Javier Cano-Sánchez, Agustín Aibar-Almazán, Yolanda Castellote-Caballero, María Aurora Mesas-Aróstegui, Andrés García-Gutiérrez, Fidel Hita-Contreras

**Affiliations:** 1 Department of Health Sciences, Faculty of Health Sciences, University of Jaén, 23071 Jaén, Spain; 2 Department of Education and Psychology, Faculty of Social Sciences, University of Atlántico Medio, 35017 Las Palmas de Gran Canaria, Spain; 3 Department of Health Sciences, Faculty of Health Sciences, University of Atlántico Medio, 35017 Las Palmas de Gran Canaria, Spain; 4 Pediatric Endocrinology Department, Instituto Hispalense de Pediatría, Hospital Quirón Marbella, 29603 Málaga, Spain; 5 Pediatrics Department, Hospital of Guadix, 18500 Granada, Spain

**Keywords:** mild cognitive impairment, physical exercise, cognitive training, systematic review, meta-analysis

## Abstract

**Background:** Mild cognitive impairment (MCI) represents an early stage of cognitive loss that significantly increases the risk of dementia. The aim of this study was to comprehensively synthesize the current evidence on the effect of combined physical and cognitive therapies in older adults with MCI. **Methods:** A systematic review with meta-analysis was conducted by searching for specific keywords in the PubMed, Scopus, Cinhal, and Web of Science databases. This meta-analysis included a total of 2256 participants distributed across 21 studies that evaluated the benefits of combining physical exercise with cognitive stimulation. **Results:** This review revealed that these types of therapies present a significant improvement in memory, attention, and executive functions. Participants showed notable improvements in these cognitive areas, highlighting the synergistic effects of physical exercise and cognitive stimulation, which exceeded the benefits of each therapy separately. These results contribute to the understanding of how these combined therapies can improve cognitive health in this population, offering robust evidence supporting their application in clinical practice. **Conclusions:** This meta-analysis shows that combined physical exercise and cognitive stimulation interventions may be an effective strategy for improving cognitive health in older adults with MCI. The findings of this study offer a valuable contribution to the field, highlighting the potential of these combined therapies to prevent cognitive decline and improve the quality of life of this population. The results may be of interest to health professionals and guide future research and clinical applications.

## 1. Introduction

Population aging is a global phenomenon that poses numerous challenges in terms of public health, especially in relation to the increase in cognitive and neurodegenerative disorders in older people [1]. One of these disorders, mild cognitive impairment (MCI), is considered an intermediate phase between normal aging and dementia and is widely recognized as an important risk factor for the development of Alzheimer’s disease and other forms of dementia [2,3]. MCI is characterized by a decline in cognitive abilities, such as memory, attention, and executive functions, without significantly affecting the activities of daily living [4]. The most frequent clinical symptom of MCI is episodic memory impairment, characterized by an accelerated rate of forgetting and difficulties in delayed recall. However, deficits in working memory (WM) and executive functions are also regularly present in individuals with MCI [5]. The first diagnostic criteria for MCI were proposed by Petersen et al. [6], focusing mainly on memory problems. These criteria included memory complaints, the preservation of daily activities, normal general cognitive function, abnormal memory for the patient’s age, and the absence of signs of dementia. However, given the identification of other non-memory problems that may also influence cognitive decline, they revised these criteria by incorporating non-memory aspects [7]. Various tests have been proposed to detect MCI, such as the Mini-Mental State Examination (MMSE), the Montreal Cognitive Assessment (MoCA), the Free and Cued Selective Recall Test (FCSRT), the California Verbal Learning Test, and the Boston Naming Test. However, no specific test or cut-off score has been established that is universally accepted for the diagnosis of MCI. The absence of standardized tests and clear cut-off points may affect the accuracy of the diagnosis [8,9].

The prevalence of MCI in people aged 65 years or older ranges between 10% and 20%, increasing with age [10]. In Spain, the prevalence of MCI is 18.5%, with a significantly higher rate in women than in men. Furthermore, this prevalence shows a notable increase with age, reaching 45.3% in individuals over 85 years of age [11]. In light of the growing elderly population and the significant impact of MCI on quality of life, as well as associated healthcare costs, there is an urgent need for interventions that can mitigate or even reverse cognitive decline in this population [12].

Non-pharmacological interventions have garnered interest as effective and safe alternatives for the management of MCI, avoiding the common side effects of pharmacological treatments [13]. Among these, combined physical and cognitive therapies have shown promise. Separately, physical exercise has been shown to effectively prevent and manage MCI, offering benefits such as fewer side effects and greater adherence than pharmacological treatments [14]. However, the literature on cognitive training often presents methodological limitations, such as small sample sizes and uncontrolled designs, which affect the reliability of its conclusions [15]. Although one study shows improvements in specific trained cognitive domains, evidence of these benefits transferring to other functional areas or daily life remains insufficient, which casts doubt on the real effectiveness of the interventions [16]. The combination of cognitive tasks during physical exercise has been shown to enhance these benefits, highlighting the synergy between physical activity and cognitive stimulation [17]. These interventions integrate physical exercise programs (such as walking, stretching, and resistance training) with cognitive stimulation activities (such as memory exercises, attention skill training, and the use of digital technologies) to optimize both physical and cognitive health [18]. It has been postulated that the combination of physical exercise and cognitive stimulation can produce synergistic effects by promoting neuroplasticity and improving cerebral blood flow, two mechanisms that are considered beneficial for maintaining cognitive health, and these results have been found in healthy older populations [19]. Furthermore, these interventions have shown additional benefits in terms of mobility, balance, and strength, key factors in reducing the risk of falls and improving functionality in daily life [20].

In this context, access to integrated interventions faces challenges related to accessibility and personalization [21]. Adapting these programs to meet individual needs, considering factors such as health status, level of cognitive impairment, and personal preferences, could maximize their effectiveness [22]. Likewise, incorporating simple and accessible technological tools, such as mobile applications or online platforms, could facilitate their implementation, especially in areas with limited resources [23].

At a conceptual level, physical exercise improves cardiovascular health and cerebral blood flow, both related to improvements in cognitive status, while cognitive stimulation strengthens neural networks and keeps critical mental functions active [24,25]. The interaction between both components could enhance the individual effects of each one, generating a synergy that favors the recovery or maintenance of cognitive functions in people with MCI [26,27]. This synergy hypothesis is supported by the literature, which suggests that combined interventions can overcome the benefits of single-component interventions. However, research on these combined therapies shows inconsistent results, which may be due to factors such as the duration of the intervention, the frequency of sessions, the specific type of activities used, and the assessment methodologies [28,29]. In addition, individual differences, such as health status, level of cognitive impairment, and lifestyle, can significantly influence the effectiveness of these therapies [30].

In this context, the present systematic review and meta-analysis aims to comprehensively synthesize the current evidence on the effect of combined physical and cognitive therapies on older people with MCI. Through a detailed analysis of recent studies, this review seeks to clarify the benefits of these interventions and determine the key components influencing their effectiveness.

## 2. Materials and Methods

This review was conducted following the guidelines of the 2020 PRISMA statement [31] and the pre-established protocol registered in PROSPERO (CRD42024620751). Furthermore, the methodological approach adhered to the recommendations outlined in the “Cochrane Manual for the Elaboration of Systematic Reviews of Interventions” [32].

### 2.1. Sources of Information

A bibliographic search was performed between October and November 2024 using the PubMed, Scopus, CINAHL, and Web of Science (WOS) databases.

### 2.2. Search Strategy

Different keywords were used in the following search string: (“combined physical” and “cognitive training” OR “cognitive training” OR “cognitive intervention” OR “cognitive therapy”) AND (“physical exercise” OR “physical activity” OR “physical training”) AND (“mild cognitive impairment” OR “mci”) AND (“older adults” OR “elderly” OR “aged”).

### 2.3. Inclusion Criteria

The selected articles had to meet the following criteria: (i) the studies must be randomized clinical trials (RCTs); (ii) the intervention studied must combine physical exercise and cognitive training; (iii) participants must belong to the older population (aged > 60 years); and (iv) participants must have a diagnosis of mild cognitive impairment (MCI) established using validated diagnostic tools, such as the Montreal Cognitive Assessment (MoCA), the Mini-Mental State Examination (MMSE), the Alzheimer’s Disease Assessment Scale–Cognitive Subscale (ADAS-Cog), among others.

### 2.4. Exclusion Criteria

Articles were excluded if they met any of the following criteria: (i) studies lacking a non-intervention reference group; (ii) studies not measuring the relevant study variables; (iii) the presence of other conditions, such as cancer, stroke, cardiovascular disease (CVD), lung disease, and/or kidney disease; and (iv) participants not reaching the minimum required attendance percentage at intervention program sessions.

### 2.5. Study Selection Process

The study selection process began with the elimination of duplicate entries and articles without available abstracts. Titles and abstracts were then thoroughly reviewed to eliminate those that did not meet the established eligibility criteria. Articles that passed this first phase were assessed in full text to determine their suitability for inclusion in the meta-analysis. To ensure objectivity and reduce potential bias, two authors (J.M.M.-P. and A.A.-A.) independently performed the selection. Cases where disagreements arose regarding the eligibility of a study were resolved by consultation with a third author (F.H.-C.), who offered his judgment to reach a consensus. This rigorous procedure ensured that all included studies were relevant and met the predefined criteria.

### 2.6. Data Extraction

The main variable in this study was cognitive status in older patients with mild cognitive impairment. Data extraction included information on authorship, publication year, study location, population details (sample size, age, and group allocation), study design, outcomes, measurement tools, intervention descriptions, measurement timelines, attrition rates, adverse effects, and key findings.

### 2.7. Assessment of Methodological Quality

Methodological quality was evaluated using the PEDro scale [33], which consists of an 11-item checklist. The highest possible score is 10 points, as the first item (“eligibility criteria”) is not included in the final scoring. Each item is rated as either “Yes” (1 point) or “No” (0 points). Quality levels are categorized as follows: scores between 0 and 3 indicate “Poor” quality, 4 and 5 represent “Fair” quality, 6 and 8 indicate “Good” quality, and scores above 9 are considered “Excellent” [34].

### 2.8. Analytic Decisions for Meta-Analysis

The results of the meta-analysis are summarized using a forest plot, which shows key details such as the lead author, year of publication, sample size, and individual effect sizes calculated with the Hedge index (g). This index is presented with its 95% confidence interval and corresponding *p*-value. To ensure the robustness of the results, a sensitivity analysis was performed. This analysis consisted of excluding studies that presented duplicate data, outliers, or single cases that could have affected the overall interpretation. The results of this sensitivity analysis were compared with those obtained in the full meta-analysis, allowing us to verify the stability of the findings and to assess whether the results were sensitive to the inclusion of certain studies. In the subgroup analysis, the studies were classified according to the tools used to assess global cognition (such as MoCA, MMSE, and ADAS-Cog). Separate meta-analyses were performed for each of these subgroups, allowing us to examine the variability of effects within each category and gain a more detailed understanding of how different assessment tools might influence the results. To address heterogeneity between studies, a random-effects model was used, allowing for differences between studies to be considered and for a more generalizable estimate of the effects. This was complemented by assessing heterogeneity through the Q test and I^2^, which provide information on the variability of effects across included studies. A high I^2^ value indicates greater heterogeneity, suggesting that studies are not homogeneous and may be influenced by different factors. In addition, publication bias was assessed using a funnel plot. This plot helps to detect potential biases related to studies reporting only positive or significant results, which could affect the validity of the meta-analysis. A visual assessment of the funnel plot and complementary statistical tests (such as Egger’s test) provided information on possible bias in the reviewed literature.

## 3. Results

### 3.1. Study Selection Process

**Figure 1:**
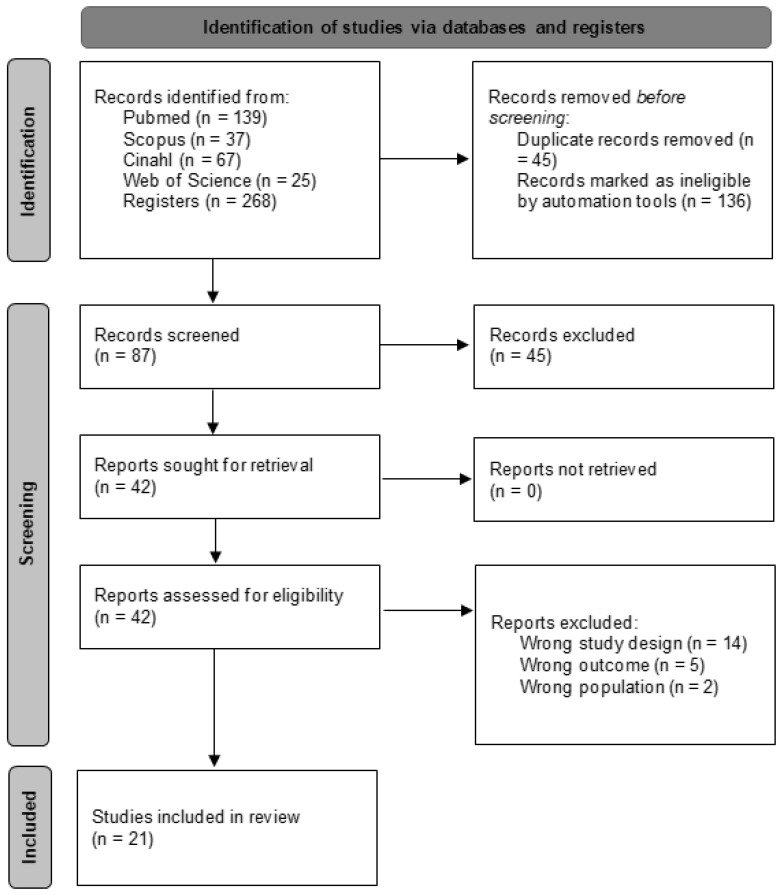
Study selection process flow chart.

An initial search across various databases identified 268 articles. The search was then refined within the same databases by focusing on specific document types (articles and randomized clinical trials) and filtering for keywords in titles and abstracts, while also eliminating duplicates. This refinement yielded 87 unique articles. These articles were subsequently screened based on their titles and abstracts, narrowing the selection to 42 potential candidates for qualitative evaluation. Ultimately, 21 articles [35,36,37,38,39,40,41,42,43,44,45,46,47,48,49,50,51,52,53,54,55] met the inclusion criteria and were included in the meta-analysis, while the remaining 21 were excluded. The selection process is outlined in greater detail in Figure 1.

### 3.2. Methodological Quality

The methodological quality of the included studies was evaluated using the PEDro scale, with the scores sourced from the PEDro web portal. Of the included studies, eighteen were classified as “Good” [35,36,37,38,40,41,42,43,44,46,47,49,50,51,52,53,54,55], while three were rated as “Fair” [39,45,48]. To ensure objectivity and consistency in the assessment, two independent reviewers scored the studies according to the PEDro scale. In cases of discrepancies between the reviewers, a conflict resolution procedure was followed. The reviewers discussed the disagreements and, if a consensus was not reached, the intervention of a third reviewer was requested. This process made it possible to ensure that the grading of the studies was as accurate and reliable as possible. A detailed assessment of the methodological quality is provided in Table 1.

### 3.3. Characteristics of the Studies

All studies included in this systematic review and meta-analysis were randomized controlled trials conducted in New Zealand [35], Italy [36], Canada [37,52], Spain [38,44], Iran [39], Taiwan [40,55], Japan [41], China [42,43,46,49,50], Greece [45], Slovakia [47], Australia [48], Turkey [51], the United States [53], and the Philippines [54]. Across these studies, 2256 participants were involved, with 967 in the control group and 1289 in the intervention group, which focused on mind–body training. Women constituted the majority in most of the included studies, with Damirchi et al. [39] reporting an entirely female sample. The overall mean age of participants was 72.4 (Table 2).

Considering these interventions, there was heterogeneity in terms of the training pace and dose employed. Regarding frequency, only one study [41] conducted therapy with one session per week. Six studies [35,38,43,46,47] carried out interventions with two weekly sessions, with the particular case of the study by Mavros et al. [48], where the frequency decreased from three sessions per week to two. Eleven trials [36,37,39,40,44,49,51,52,53,54,55] implemented three weekly sessions. Finally, in three articles [42,45,50], the intervention was applied five days a week.

On the other hand, in other studies [37,45,52], the sample was randomized into five groups. In four other studies [39,48,51,54], four treatment groups were used, while Xu et al. [49] employed three groups.

### 3.4. Study Results

Of the 21 articles included in this systematic review, five were excluded from the meta-analysis. The article by Bray et al. [47] was excluded because it did not evaluate global cognition but rather assessed changes in functional brain connectivity. A similar situation occurs with [39,40,53], which assessed specific components of executive function, such as attention, decision-making capacity, processing speed, or memory. A separate case is the article by Lipardo et al. [54], which was excluded for not examining cognition.

The main objective of this systematic review and meta-analysis was to evaluate global cognition. Regarding this evaluation, seven articles used the Montreal Cognitive Assessment (MoCA), eight studies employed the Mini-Mental State Examination (MMSE), and five trials used the Alzheimer’s Disease Assessment Scale–Cognitive Subscale (ADAS-Cog). Among the studies assessing global cognition, 10 reported significant improvements supporting combined therapies [38,42,43,44,45,46,47,50,51,52], achieving a statistical value of *p* < 0.05. Significant results in cognition were also observed in the studies excluded from the meta-analysis [37,39,40,53], based on their respective scales measuring specific cognitive aspects, favoring combined training. The remaining articles [35,41,48,49,55] did not reach statistical significance (*p* > 0.05) when measuring changes in global cognition after this type of training.

In this systematic review, secondary variables such as physical function, depression, and quality of life are also considered. Physical function was evaluated in thirteen trials [35,37,38,40,43,46,47,48,49,50,51,53,54] using scales such as the 6-Minute Walk Test (6MWT), the Timed Up and Go (TUG) Test, the Sit to Stand Test (STS), the 30-Second Chair Stand Test, and the Short Physical Performance Battery (SPPB), which were among the most frequently used. Statistically significant differences favoring combined training were found in most articles [35,38,40,46,47,48,50,51,53,54], while the remaining studies [37,43,49] did not conclude significant changes.

Additionally, depression was studied in four trials [35,46,49,51] using the Hospital Anxiety and Depression Scale (HADS), the Geriatric Depression Scale (GDS), and the Hamilton Depression Rating Scale (HDRS), with significant improvements observed in almost all included studies [35,46,51]. However, this was not the case for Xu et al. [49], where the GDS results did not show significant differences between groups.

Finally, quality of life was analyzed in five of the included studies [35,46,47,49,51], and like for the previous variable, all studies except Xu et al. [49] achieved significant improvements in quality of life. This was evaluated using scales such as the Quality of Life in Alzheimer’s Disease Scale, the Quality of Life Test, the EuroQoL 5-D Questionnaire (EQ-5D), and the World Health Organization Quality of Life Old Module.

### 3.5. Meta-Analysis

**Figure 2:**
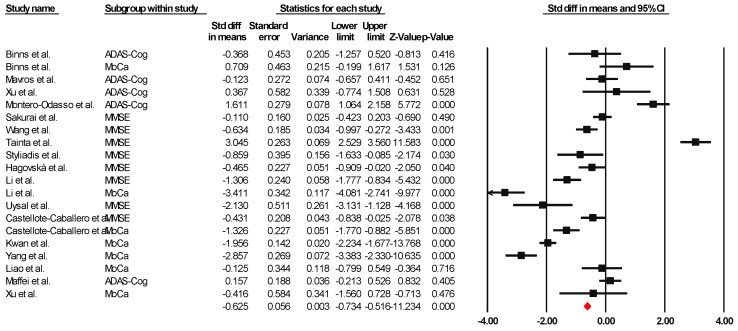
Forest plot of the overall effect of combined physical and cognitive therapies for the health of older adults with mild cognitive impairment. The black box represents the point estimate for each study, while the box size represents the population size, and the horizontal line is the 95% CI. The diamond-shaped figure represents the estimated point of the mean difference [35,36,38,40,41,42,43,44,45,46,47,48,49,50,51,52].

Sixteen of the articles were integrated into the meta-analysis to synthesize the findings on global cognition. The heterogeneity analysis showed that the Q-value was 8.418 with fourteen degrees of freedom. The I-squared statistic, which quantifies the percentage of variability in the observed effects attributed to real effects rather than sampling error, was set to 0%. In addition, we calculated Tau-squared and Tau, which provided information on the variance and standard deviation of the real effect sizes in d units, respectively. Both Tau-squared and Tau were calculated as 0.000, suggesting that all studies shared a common effect size without any dispersion of real effects. Lastly, the prediction interval was not reported because our analysis estimated Tau-squared as zero, reinforcing the notion that all studies exhibited consistent effect sizes without any variability in real effects. This comprehensive analysis incorporating these statistical measures provides valuable information about the results and the observed homogeneity in the effects across the selected studies. Since the I-square, Tau-square, and Tau indices of heterogeneity are minimal, a fixed-effect model was used for the analysis. The effect size index used was the standardized difference between means (g), −0.625, with a 95% confidence interval of −0.734 to −0.516. Importantly, negative effect size values represent an improvement in global cognition attributable to the combined interventions, indicating their success. Figure 2 presents this result through a graph illustrating the consistency of the effects observed across the included studies.

#### 3.5.1. Subgroup Analysis

A subgroup analysis was performed using the three global cognition measurement tools. The results revealed notable statistical significance, supported by moderate and inversely negative Hedge’s g effect sizes. Subgroup analyses based on this assessment tool demonstrated consistent effect sizes across all cases. This consistency in our results suggests that the choice of assessment tool had a minimal impact on the observed treatment effects.

##### MoCa

**Figure 3:**
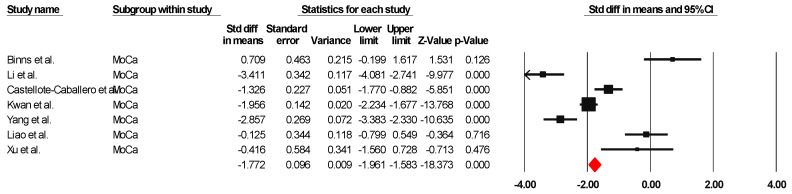
Forest plot of the effectiveness of combined physical and cognitive therapies for the health of older adults with mild cognitive impairment [35,38,40,43,46,49,50].

**Figure 4:**
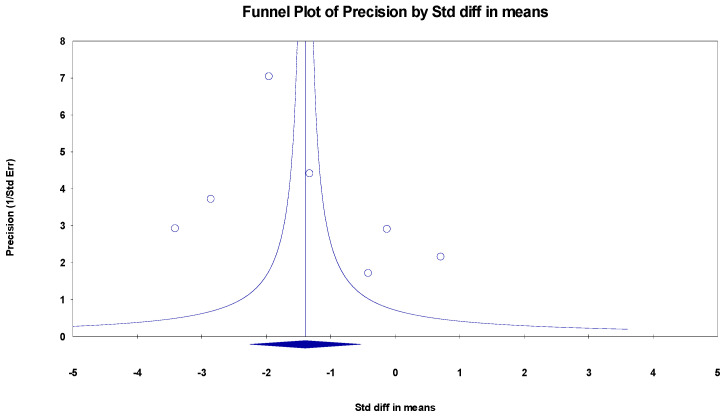
Funnel plot for MoCa.

The results indicated an effect size of −1.774 for the MoCa, showing the substantial impact of this measurement tool on the observed outcomes. Independent Q tests were performed, showing evidence of significant heterogeneity, as the Q-value was 5.802 with three degrees of freedom (df) and a *p*-value of 0.000 (Figure 3), without risk of publication bias (Egger *p* = 0.20) (Figure 4).

##### MMSE

**Figure 5:**
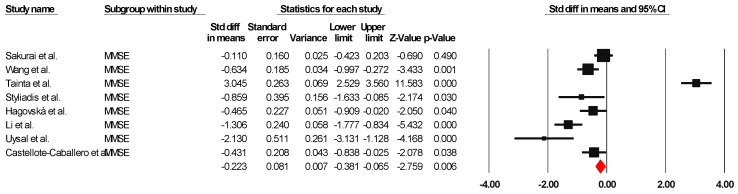
Forest plot of the effectiveness of combined physical and cognitive therapies for the health of older adults with mild cognitive impairment [38,41,42,44,45,47,50,51].

**Figure 6:**
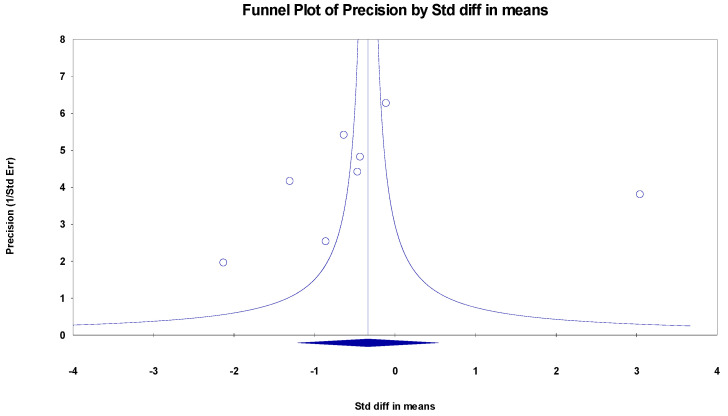
Funnel plot for MMSE.

The results indicated an effect size of −0.187 for the MMSE, showing the substantial impact of this measurement tool on the observed outcomes. Independent Q tests were performed, showing evidence of significant heterogeneity, as the Q-value was 7.587 with six degrees of freedom (df) and a *p*-value of 0.000 (Figure 5), without risk of publication bias (Egger *p* = 0.42) (Figure 6).

##### ADAS-Cog

**Figure 7:**
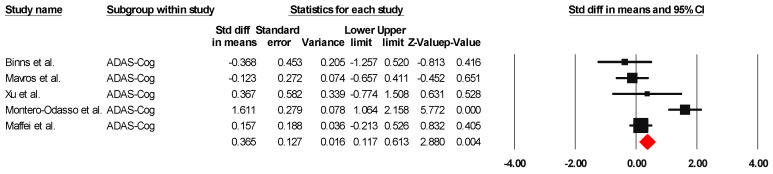
The forest plot of the effectiveness of combined physical and cognitive therapies for the health of older adults with mild cognitive impairment [35,36,48,49,52].

**Figure 8:**
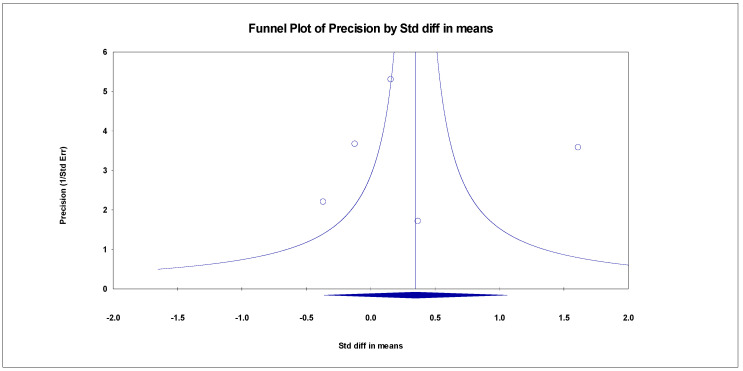
Funnel plot for ADAS-Cog.

The results indicated an effect size of 0.518 for the ADAS-Cog, showing the substantial impact of this measurement tool on the observed outcomes. Independent Q tests were performed, showing evidence of significant heterogeneity, as the Q value was 4.978 with three degrees of freedom (df) and a *p*-value of 0.000 (Figure 7), without risk of publication bias (Egger *p* = 0.49) (Figure 8).

## 4. Discussion

The main objective of this study was to evaluate the effectiveness of combining physical exercise and cognitive training in improving cognitive performance in older adults with mild cognitive impairment (MCI). Through a meta-analysis of the available randomized clinical trials, we sought to determine whether this combined intervention could generate significant benefits compared with traditional treatments or individual interventions, as well as to evaluate the sustainability of these effects in the long term.

In terms of methodological quality, most of the studies analyzed [35,36,37,38,40,41,42,43,44,46,47,49,50,51,52,53,54,55] showed good quality in design and execution. However, three [39,45,48] presented an average methodological quality. None of the included studies achieved an excellent rating. Notably, the main limitations in these studies were a lack of blinding of participants or therapists and an inadequate assignment of interventions. These methodological deficiencies may have significantly impacted the observed results. Previous research has shown that a lack of blinding and an inadequate assignment of tasks can increase the possibility of results being exaggerated by up to 13% and 7%, respectively [56]. Furthermore, one aspect that should be noted in the reviewed studies is a lack of detailed analysis isolating the specific effects of physical exercise and cognitive training. Although the combination of both interventions is the main hypothesis of this review, some studies may not have separately evaluated the effects of each component due to the inherent complexity of intervention designs. However, this should not be interpreted as a flaw in the design but rather as an inherent characteristic of multifaceted interventions, in which the interaction between multiple components is sought to be exploited to obtain global benefits. The fact that the effects of each intervention cannot be broken down independently does not invalidate the results obtained but rather reflects the integrative nature of combined therapies. Indeed, many clinical interventions specifically seek synergistic effects that cannot be observed by implementing isolated interventions. Furthermore, in the context of MCI, the combined approach may be more representative of real interventions, which typically include both physical exercise and cognitive training together. Despite the absence of a detailed analysis of the separate effects, the relevance of this work lies in its ability to provide empirical evidence on the efficacy of combined therapies in a realistic context. Rather than focusing solely on isolated components, this approach reflects a strategy closer to clinical and rehabilitation practices, where the aim is to improve the overall well-being of patients by combining various therapeutic modalities.

Several recent systematic reviews have evaluated the effects of physical exercise on cognitive function in older adults, both with and without cognitive deficits. In general, these reviews have found that physical exercise can have positive effects on different cognitive domains. However, some studies have focused exclusively on people with MCI. In this regard, more recent research has documented that physical exercise improves global cognitive function in older adults with MCI, specifically in areas such as working memory and executive cognition [57,58,59,60,61]. Although the results have been promising, some studies have included samples that were not diagnosed with MCI, which could influence the generalizability of the observed effects [58,59,60,61,62].

Despite advances, there are still aspects to be evaluated. In particular, several studies have not considered important moderating factors, such as duration, modality, and intensity of physical exercise, limiting our understanding of the specific mechanisms underlying the observed cognitive benefits [57,61]. Indeed, previous meta-analyses have shown that physical exercise has a more robust effect than pharmacological treatments in improving cognition in patients with MCI, with longer interventions (at least six months) showing greater benefits; however, these studies did not analyze key parameters such as intensity and type of exercise in detail, which is crucial to understanding how physical exercise influences cognitive function [61,63]. That said, some reviews have indicated that, although physical exercise improves global cognitive function in older adults with MCI, it does not necessarily significantly affect other cognitive domains [60,64]. Recently, the work of Law et al. [59] demonstrated that the positive effects of physical exercise on cognition could be especially related to working memory, suggesting that this domain is the most sensitive to physical interventions in older people with MCI. Low-impact physical interventions, such as walking or tai chi, have also shown positive effects on the cognition of older adults, especially in terms of reducing the risk of cognitive decline and improving overall quality of life [65,66]. These approaches, although valuable, tend to offer more limited benefits than more intensive or combined exercise and cognitive stimulation programs [67].

However, cognitive training has also shown positive effects on cognitive function in individuals with MCI. Previous reviews such as that of Wei et al. [68] have shown that cognitive training significantly benefits general cognitive function in individuals with cognitive impairment, as well as other cognitive variables, such as attention, orientation, delayed memory, and language skills. Similarly, Lampit et al. [69], in their systematic review, documented that cognitive behavioral therapy has moderate effectiveness in improving cognitive abilities, albeit in healthy older adults.

Furthermore, cognitive training through games, mental tasks, or even reminiscence therapy has been shown to offer improvements in memory, processing speed, and attention span in older adults with mild cognitive impairment [70,71]. These cognitive stimulation programs are often accessible and relatively easy to implement, making them valuable in both clinical and community settings. However, while these cognitive stimulation programs can be effective, the reviewed studies suggest that the combination of physical exercise and cognitive training is even more effective, as it amplifies the benefits of each intervention separately [54,72]. This combination significantly impacts the cognitive areas most affected in older adults with MCI, such as memory, attention, and executive functions. Physical exercise can help maintain neuroplasticity, providing a solid foundation on which cognitive training can work more effectively [73].

Combined interventions integrating moderate physical exercise and cognitive training have emerged as some of the most promising strategies to address MCI. The results of this systematic review and meta-analysis confirm that combined physical exercise and cognitive training interventions offer significant benefits for improving global cognition, supporting previous findings from other systematic reviews such as that by Reiker et al. [19], in which they observed improvements in cognitive functioning after combined cognitive and physical interventions; however, unlike our study, these interventions were carried out in healthy older adults. Our findings also support those of Lauenroth et al. [74], who analyzed studies with samples ranging from healthy older adults to people with Alzheimer’s and concluded that, although it seems that combined training positively influences cognition, due to the heterogeneity of studies with respect to a series of vital methodological parameters, such results should be interpreted with caution.

In our analysis, 10 of the 21 articles that assessed global cognition using scales such as MoCA, MMSE, or ADAS-Cog reported significant improvements (*p* < 0.05), which is consistent with the evidence that these multifaceted strategies improve key cognitive functions such as memory and executive functions. Furthermore, although not included in the meta-analysis because they sampled healthy older adults, studies that assessed specific aspects of cognition also favor combined interventions. This reinforces the findings of Castaño et al. [75], who showed that resistance training combined with cognitive training increases brain-derived neurotrophic factor and improves cognitive function.

Furthermore, although not all studies in this systematic review managed to achieve statistical significance in global cognition, the positive results observed in secondary variables such as physical function, quality of life, and depression add value to the comprehensive approach of these interventions. Most studies on physical function, for example, show significant improvements with scales such as the 6MWT, TUG, and SPPB. This supports the claim of Aminirakan et al. [76], who argued that these interventions not only improve cognitive aspects but also optimize functional capacities, which is essential for the daily life of patients with MCI.

Regarding depression and quality of life, the results are consistent with the review by Karssemeijer et al. [77], which noted that combined programs reduce symptoms of depression and promote improvements in psychological well-being. In the present review, three of the four studies assessing depression found significant differences, and four of five studies on quality of life also demonstrated notable benefits. These improvements could be due, in part, to the positive impact of physical exercise on the hippocampus–amygdala axis and emotional responses, as well as to cognitive stimulation, which promotes greater control and sense of purpose in patients. This is particularly relevant for healthcare professionals working with older adults at risk of dementia, as it suggests that a holistic approach that addresses both the body and mind offers amplified benefits.

Despite the positive results, this meta-analysis has several limitations that need to be considered. First, the heterogeneity of the included studies, both in terms of the types of exercise and cognitive tasks used, makes it difficult to generalize the results. Some studies used brief or low-intensity interventions, which could have limited the magnitude of the observed effects. Furthermore, the methodological quality of the included studies varied considerably, introducing a possible bias in the final results. Future studies should use more homogeneous protocols and better control for confounding variables, such as the initial level of cognition and the presence of comorbidities that could affect the results.

## 5. Conclusions

This review and meta-analysis provides strong evidence that combined physical and cognitive exercise therapy is an effective strategy for improving cognition in older adults with MCI. However, further research with high-quality studies using standardized protocols is needed to better understand the underlying mechanisms and optimize these interventions. It is also crucial to consider individual variations in participant characteristics, such as prior physical activity levels and comorbidities, to design more personalized interventions. The results of this meta-analysis support the idea that combined interventions have significant therapeutic potential, which can be a useful tool in preventing and managing cognitive decline in the aging population.

## Figures and Tables

**Table 1:** Methodological quality of the included articles.

	1	2	3	4	5	6	7	8	9	10	11	Total Score
Binns et al. [35]	1	1	1	1	0	0	1	1	0	1	1	7
Maffei et al. [36]	1	1	0	1	0	0	1	1	1	1	1	7
Bray et al. [37]	1	1	0	1	1	0	1	1	0	1	0	6
Castellote-Caballero et al. [38]	1	1	1	1	0	0	0	1	1	1	1	7
Damirchi et al. [39]	1	1	0	1	0	0	0	0	0	1	1	4
Liao et al. [40]	1	1	1	1	0	0	1	0	0	1	1	6
Sakurai et al. [41]	0	1	0	1	0	0	1	0	1	1	1	6
Wang et al. [42]	1	1	0	1	0	0	1	1	1	1	1	7
Kwan et al. [43]	1	1	1	1	0	0	1	1	1	1	1	8
Tainta et al. [44]	1	1	1	1	1	0	0	1	1	1	1	8
Styliadis et al. [45]	1	1	0	1	0	0	0	1	0	1	1	5
Yang et al. [46]	1	1	1	1	0	0	0	1	1	1	1	7
Hagovská et al. [47]	1	1	1	1	1	0	1	1	0	1	1	8
Mavros et al. [48]	0	1	0	1	0	0	0	1	0	1	1	5
Xu et al. [49]	1	1	1	1	0	0	1	1	0	1	1	7
Li et al. [50]	1	1	1	1	0	0	1	1	0	1	1	7
Uysal et al. [51]	1	1	1	1	0	1	1	1	0	1	1	8
Montero-Odasso et al. [52]	1	1	1	1	1	0	1	0	1	1	1	8
Fairchild et al. [53]	1	1	1	1	1	0	0	1	0	1	1	7
Lipardo et al. [54]	1	1	1	1	0	0	1	0	1	1	1	7
Liao et al. [55]	1	1	1	1	0	0	1	0	0	1	1	6

Items: 1: eligibility criteria; 2: random allocation; 3: concealed allocation; 4: baseline comparability; 5: blind subjects; 6: blind therapists; 7: blind assessors; 8: adequate follow-up; 9: intention-to-treat analysis; 10: between-group comparisons; 11: point estimates and variability; yes = 1; no = 0.

**Table 2:** Characteristics of the included studies.

Author and Year	Sex	Sample CG/IG	Control Group	Intervention Group			
				Age	Treatment	Exercise Parameters	Results
Binns et al. [35]	F: 74.5% M: 25.5%	9/11	Cognitive stimulation therapy	85.6	Cognitive therapy Aerobics Strength Balance	F: 2 times/week #S: 14 sessions D: 60 min	Thirty-six residents were screened, with twenty-three participants randomized to intervention (CogEx, n = 10) or control (CST, n = 13) groups. The assessments took 45 min to 1.5 h, and there was repetition between the two cognitive measures. Ten facilitators completed training with the manualized program. Exercises were combined into an hour-long CST session; however, limited balance training occurred, with participants exercising predominantly in sitting positions. The facilitators felt the participants engaged more and were safer sitting.
Maffei et al. [36]	F: 47.8% M: 52.2%	58/55	Usual life routine	74.5 ± 4.6	Cognitive training Physical exercise Music therapy	F: 3 times/week #S: 84 sessions D: 60 min	The significant beneficial effect of combined training on the ADAS-Cog was detected (*p* = 0.007) in the MCI-training group and (*p* = 0.026) in the MCI-no training group. The difference between groups was statistically significant (*p* < 0.0001); training increased parahippocampal CBF, but no effect on GM volume loss was evident. Increased BOLD activity, indicative of decreased neuronal efficiency, was found only in untrained MCI subjects.
Bray et al. [37]	F: 47.8% M: 52.2%	17/73	Physical exercise control Cognitive training control Vitamin D control	73.9 ± 6.5	Physical exercise Cognitive training Vitamin D	F: 3 times/week #S: 60 sessions D: 90 min	In the FBC region of interest, there was a significant between-arm difference in T0 Salience Network connectivity in model four. The intervention arm demonstrated a significant between-arm increase (T6–T0) in connectivity for a single cluster in model four (*p-FDR* < 0.05). On the right hippocampus, intervention arms demonstrated a significant between-arm increase (T6–T0) in model one (*p-FDR* < 0.01 and <0.05), two (*p-FDR* = <0.001), and four (*p-FDR* = <0.01). On the left hippocampus, there was a significant between-arm difference in connectivity with the left inferior frontal and precentral gyrus at T0 in model four. The intervention arm demonstrated a significant between-arm increase (T6–T0) in only model four (*p-FDR* < 0.01).
Castellote-Caballero et al. [38]	F: 77.7% M: 22.3%	47/48	Cognitive stimulation	72.1 ± 4.25	Cognitive stimulation Psychomotor sessions	F: 2 times/week #S: 24 sessions D: 45–50 min	The results show significant improvements in both aspects, such as balance (*p* = 0.035), gait (*p* = 0.001), upper and lower body strength (*p* = 0.000 and *p* = 0.001), flexibility (*p* = 0.000), physical function (*p* = 0.001), cognitive function (*p* = 0.041), cognitive impairment (*p* = 0.000), verbal fluency (*p* = 0.000), and executive functions (*p* = 0.000) in the group that carried out the intervention compared with the control group.
Damirchi et al. [39]	F: 100%	9/35	Waitlist	68.4	Physical training Mental training Combined training	F: 3 times/week #S: 24 sessions D: 30–60 min	Analysis of variance with Tukey post hoc test revealed a significant increase in working memory (*p* = 0.012) and brain-derived neurotrophic factor (*p* = 0.024) in the ME group compared with the control group. Furthermore, compared with the physical training group, the ME group demonstrated better working memory (*p* = 0.014) and processing speed (*p* = 0.024).
Liao et al. [40]	F: 67.7% M: 32.3%	16/18	Combined physical and cognitive training	74.3	VR-combined physical and cognitive training	F: 3 times/week #S: 36 sessions D: 30–60 min	Both groups significantly improved in the SCWT and the single-task and motor dual-task gait performance measures. However, only the VR group showed improvements in cognitive dual-task gait performance and the DTC of cadence. Moreover, the VR group showed more improvements than the CPC group in the TMT-B and DTC of cadence with borderline significance.
Sakurai et al. [41]	F: 52% M: 48%	218/215	General health-related information	74.4	Management of vascular risk factors Exercise Nutritional counseling Cognitive training	F: 1 time/week #S: 78 sessions D: 90 min	The between-group difference in composite score changes was 0.047 (95% CI: −0.029 to 0.124) for cognitive tests. Secondary analyses indicated positive impacts of interventions on several secondary health outcomes. The interventions appeared to be particularly effective for individuals with high attendance during exercise sessions and those with the apolipoprotein E *ε*4 allele and elevated plasma glial fibrillary acidic protein levels.
Wang et al. [42]	F: 60% M: 40%	61/62	Routine health education program	67.1	Cognitive training (mnemonic strategy training) Lifestyle guidance intervention (diet, sleep, and exercise guidance)	F: 5 times/week #S: 45 sessions D: 20–90 min	For cognitive outcomes, the linear mixed-effect model results showed significant time × group effects in the MMSE (Cohen *d* = 0.63 [95% CI, 0.27 to 1.00], *F* = 10.25, *p* = 0.002). This study found significant time × group effects in AVLT-immediate (Cohen *d* = 0.47 [95% CI, 0.11 to 0.83], *F* = 8.18, *p* = 0.005), AVLT-delayed (Cohen *d* = 0.45 [95% CI, 0.10 to 0.81], *F* = 4.59, *p* = 0.034), LMT-delayed (Cohen *d* = 0.71 [95% CI, 0.34 to 1.07], *F* = 4.59, *p* = 0.034), DSST (Cohen *d* = 0.27 [95% CI, −0.08 to 0.63], *F* = 4.83, *p* = 0.030), and DST (Cohen *d* = 0.69 [95% CI, 0.33 to 1.05], *F* = 8.58, *p* = 0.004).
Kwan et al. [43]	F: 78.2% M: 22.8%	147/146	Usual care	74.5 ± 6.8	VR motor–cognitive training (VRMCT)	F: 2 times/week #S: 16 sessions D: 60 min	VRMCT was effective in promoting global cognitive function (interaction effect: *p* = 0.03), marginally promoting executive function (interaction effect: *p* = 0.07), and reducing frailty (interaction effect: *p* = 0.03). The effects were not statistically significant on other outcomes.
Tainta et al. [44]	F: 58% M: 42%	64/61	Regular health advice	75.6 ± 6.5	Cardiovascular risk factor monitoring Nutritional workshops Cognitive stimulation and training Physical exercise	F: 3 times/week #S: 144 sessions D: 90 min	More than 70% of the participants had high overall adherence to the intervention activities. The risk of cognitive decline was higher in the RHA group than in the MD-Int group in terms of executive function (*p* = 0.019) and processing speed scores (*p* = 0.026).
Styliadis et al. [45]	F: 64.3% M: 35.7%	28/42	Active control group (training protocol consisting of watching a documentary and answering a questionnaire) Passive control group (participants did not engage in any activity)	70.8 ± 5.7	Combined physical and cognitive training	F: 5 times/week #S: 40 sessions D: 60 min	A significant training effect was identified only after the combined training scheme: a decrease in the post- compared with the pre-training activity of the precuneus/posterior cingulate cortex in delta, theta, and beta bands. This effect was correlated to improvements in cognitive capacity as evaluated by MMSE scores [(score difference in delta (*p* = 0.043) and theta bands (*p* = 0.015)]. The results indicate this type of training shows indices of a positive neuroplastic effect in MCI patients and that EEG may serve as an index of gains versus cognitive declines and neurodegeneration.
Yang et al. [46]	F: 52.7% M: 47.3%	57/55	Usual care	70.2 ± 6.0	Dietary intervention Physical training Cognitive training Monitoring of metabolic indicators and vascular risk factors	F: 2 times/week #S: 47 sessions D: 60–90 min	At baseline, clinical characteristics did not differ significantly between groups. Significant interaction effects between time and group were detected (*p* < 0.001), indicating that the scores of five outcomes (cognitive function, short physical performance battery, Timed Up and Go Test, quality of life, and depression) of the intervention and control groups changed differently over time. Participants in the intervention group had a significantly greater improvement in cognitive function, physical function, and quality of life and fewer depression symptoms compared with the control group at baseline and follow-up periods.
Hagovskà et al. [47]	F: 48.7% M: 51.3%	40/40	Balance training	67.1	CogniPlus training program Balance training	F: 2 times/week #S: 20 sessions D: 30 min	The two groups showed significant differences recorded after training in the Mini-Mental State Examination. Before the training, there were no significant differences recorded between the groups in global cognitive functions as assessed by the MMSE. After the training, there were significant differences in favor of the experimental group (*p* < 0.05). The Timed Up and Go Test with dual tasking, balanced by the Tinetti test, demonstrated the quality of life in favor of the experimental group (*p* < 0.03–0.001). There were no significant differences between the groups in assessing fear of falling or other monitored parameters.
Mavros et al. [48]	F: 68% M: 32%	27/73	Sham cognitive training Sham progressive resistance training	Aged ≥ 55	Cognitive training Progressive resistance training	F: 3 to 2 times/week #S: 58 sessions D: 60–100 min	PRT increased upper (standardized mean difference (SMD) = 0.69, 95% confidence interval = 0.47, 0.91), lower (SMD = 0.94, 95% CI = 0.69–1.20), and whole-body (SMD = 0.84, 95% CI = 0.62–1.05) strength, and percentage change in VO_2peak_ (8.0%, 95% CI = 2.2–13.8) was significantly higher than sham exercise. Higher strength scores, but not greater VO_2peak_, were significantly associated with improvements in cognition (*p* < 0.05). Greater lower body strength significantly mediated the effect of PRT on ADAS-Cog improvements (indirect effect: b = 0.64, 95% CI = 1.38 to 0.004; direct effect: b = 0.37, 95% CI = 1.51–0.78) and global domain (indirect effect: b = 0.12, 95% CI = 0.02–0.22; direct effect: b = 0.003, 95% CI = 0.17–0.16) but not for the executive domain (indirect effect: b = 0.11, 95% CI = 0.04–0.26; direct effect: b = 0.03, 95% CI = 0.17–0.23).
Xu et al. [49]	F: 73.3% M: 26.3%	6/13	Health advice	74 ± 5.2	Cognitive training Mind–body physical exercise Nurse-led risk factor modification	F: 3 times/week #S: 36 sessions D: 30 min	Significant within-group changes were observed in HK-MoCA in RFM (4.50 ± 2.59, *p* = 0.008), cost of health service utilization in CPR (−4000, quartiles: −6800 to −200, *p* = 0.043), fish and seafood in HA (−1.10 ± 1.02, *p* = 0.047), and sugar in HA (2.69 ± 1.80, *p* = 0.015). Group × time interactions were noted in HK-MoCA favoring the RFM group (*p* = 0.000), DAD score favoring the CPR group (*p* = 0.027), GAS-20 favoring the CPR group (*p* = 0.026), number of servings of fish and seafood (*p* = 0.004), and sugar (*p* < 0.001) eaten per day.
Li et al. [50]	F: 60.7% M: 39.3%	42/42	General community health instruction	71.1	Aerobic training Strength training Balance training Coordination training Sensitivity training	F: 5 times/week #S: 120 sessions D: 30 min	The average CM-PPT score increased from 11.36 ± 2.69 to 11.88 ± 2.40 and 12.83 ± 2.19 in 3 and 6 months, respectively, after the intervention, while the control group showed a decrease from 10.79 ± 2.73 to 10.24 ± 2.62 in 3 months and 9.21 ± 2.09 in 6 months. CM-PPT scores with the main intervention effect and the interaction between intervention and time were both statistically significant (*p* < 0.05), indicating that the physical functions of participants with MCI were improved after intervention. The average MoCA score increased from 21.52 ± 2.05 to 23.48 ± 1.47 (3 months) and 25.19 ± 1.29 (6 months) after intervention, while the control group showed a decrease from 21.14 ± 1.97 to 20.21 ± 1.88 and 19.45 ± 2.00 in 3 and 6 months. The MMSE score showed the same trend as the MoCA score. The MoCA score with the main intervention effect; the MMSE and MoCA scores with the effect of time; and the MMSE and MoCA scores with the interaction between the intervention and time were all statistically significant (*p* < 0.05), showing that the cognitive function of participants with MCI was improved by the intervention.
Uysal et al. [51]	F: 16.7% M: 83.3%	12/36	Exclusively lower extremity strengthening exercises	73.7	Aerobic exercise Dual-task training Lower extremity strengthening exercise	F: 3 times/week #S: 36 sessions D: 30 min	In all three intervention groups, there was a significant improvement in cognitive status, balance, mobility, activity-specific balance confidence, physical performance, mood, and quality of life (*p* < 0.05). The most remarkable change was observed in the ADG regarding cognitive status, mobility, and physical performance parameters (*p* < 0.05). In addition, the most significant improvement in balance parameters was recorded both in the DG and ADG (*p* < 0.05). The highest increase in functional exercise capacity was detected both in the AG and the ADG (*p* < 0.05). Furthermore, both exercise combinations were superior to the control group in terms of improving mood and quality of life (*p* < 0.05).
Montero-Odasso et al. [52]	F: 49.1% M: 50.9%	34/141	Balance–toning exercise Sham cognitive training Placebo vitamin D	73.1 ± 6.6	Aerobic and resistance training Cognitive training Vitamin D	F: 3 times/week #S: 60 sessions D: 90 min	At 6 months, all active arms (i.e., arms 1 through 4) with aerobic–resistance exercise, regardless of the addition of cognitive training or vitamin D, improved ADAS-Cog-13 compared with the control (mean difference, −1.79 points; 95% CI, −3.27 to −0.31 points; *p* = 0.02; *d* = 0.64). Compared with exercise alone (arms 3 and 4), exercise and cognitive training (arms 1 and 2) improved the ADAS-Cog-13 score (mean difference, −1.45 points; 95% CI, −2.70 to −0.21 points; *p* = 0.02; *d* = 0.39). No significant improvement was found with vitamin D. Finally, the multidomain intervention (arm 1) improved the ADAS-Cog-13 score significantly compared with the control (mean difference, −2.64 points; 95% CI, −4.42 to −0.80 points; *p* = 0.005; *d* = 0.71). Changes in ADAS-Cog-Plus were not significant.
Fairchild et al. [53]	F: 4.2% M: 95.8%	36/36	Stretching exercise Cognitive training	72.4 ± 9.5	Aerobic and resistance training Cognitive training	F: 3 times/week #S: 72 sessions D: 60 min	Controlling for age and employment status, linear mixed-effects models revealed that all participants experienced significant improvement in the delayed recall of a word list, learning and memory, and executive function. Only the CARE + CT condition significantly improved processing speed and functional capacity. APOE4 status impacted the cognitive benefits of those with the SE + CT condition.
Lipardo et al. [54]	F: 79% M: 21%	23/69	Waitlist	69 ± 8.3	Physical training Cognitive training	F: 3 times/week #S: 36 sessions D: 60–90 min	No significant difference was observed across time or groups in fall incidence rate at 12 weeks (*p* = 0.152) and 36 weeks (*p* = 0.954). The groups did not statistically differ in other measures except for a significant improvement in dynamic balance based on the Timed Up and Go Test in the training group (9.0 s with *p* = 0.001) and in the cognitive training alone group (8.6 s with *p* = 0.012) compared with the waitlist group (11.1 s) at 36 weeks.
Liao et al. [55]	F: 67.6% M: 32.4%	16/18	Combined physical and cognitive training	74.3	VR-based physical and cognitive training	F: 3 times/week #S: 36 sessions D: 60 min	Both groups showed improved executive function and verbal memory (immediate recall). However, only the VR group showed significant improvements in global cognition (*p* < 0.001), verbal memory (delayed recall, *p* = 0.002), and IADL (*p* < 0.001) after the intervention. The group × time interaction effects further demonstrated that IADLs were more significantly improved with VR training than CPC training (*p* = 0.006). The hemodynamic data revealed decreased activation in prefrontal areas after training (*p* = 0.015), indicative of increased neural efficiency, in the VR-trained subjects.

F: frequency; #S: number of sessions; D: duration.

## Data Availability

Not applicable.
